# Molten Alkali-Assisted Formation of Silicate Gels and Its Application for Preparing Zeolites

**DOI:** 10.3390/gels10060392

**Published:** 2024-06-09

**Authors:** Juan Ye, Yanchun Yang, Li Zhang, Man Li, Yiling Wang, Yuxuan Chen, Ruhui Ling, Jiefeng Yan, Yan Chen, Jinxing Hu, Zhenxing Fang

**Affiliations:** 1 College of Science and Technology, Ningbo University, 521 Wenwei Road, Ningbo 315300, China; 13252058707@163.com (J.Y.); a13736170883@163.com (L.Z.); 18758807683@163.com (M.L.); 13003713750@163.com (Y.W.); cyx2918380445@163.com (Y.C.); 18142011326@163.com (R.L.); yanjiefeng@nbu.edu.cn (J.Y.); hujinxing@nbu.edu.cn (J.H.); 2 Ecological Environment Monitoring Station in Yanji City, Yanji 133001, China; yjjczyang@163.com; 3 State Key Laboratory of Inorganic Synthesis and Preparative Chemistry, Jilin University, Changchun 130012, China; yanchen@jlu.edu.cn

**Keywords:** fly ash, silicate gels, zeolites, ammonium adsorption, ion exchange

## Abstract

Fly ash was used as raw material to prepare zeolites through silicate gels, assisted by the hydrothermal method. The silicate gels could be effectively formed in a few minutes in a molten alkali environment. The zeolites could be prepared by using these silicate gels through the hydrothermal method, which realizes the transformation from useless materials to highly valuable materials. The obtained zeolites were applied to the removal of ammonium in water, achieving the highvalue utilization of fly ash. The synthesized zeolites were characterized by X-ray diffraction (XRD), scanning electron microscopy (SEM), energy dispersive spectrum (EDS), thermogravimetric (TG), and Fourier transform infrared (FTIR) spectroscopy. The study on the adsorption and removal of ammonium in water shows that the adsorption of ammonium is more in line with pseudo first-order kinetics, and the adsorption mainly occurs in the first 20 min. The adsorption can reach equilibrium in 30 min, and the maximum adsorption capacity can reach 49.1 mg/g. The adsorption capacity of ammonium has the best performance at pH = 5. Furthermore, within a certain range, an increase in temperature is beneficial for the removal of ammonium.

## 1. Introduction

Zeolite is widely used in pollutant control, petrochemistry, water purification, gas purification, and other fields because of its excellent catalytic ability, cation exchange ability, and adsorption ability [1,2,3,4]. The main chemical components of fly ash are silica and alumina, which are similar to zeolite [5,6]. The main difference among them lies in the different crystal structures. Therefore, fly ash is considered a potential raw material for synthesizing zeolite. However, the calcium oxide and some other oxides in fly ash can affect the crystal transformation of mullite and reduce the ion exchange capacity of zeolite. The residual carbon can also reduce the whiteness of zeolite products. Therefore, pre-treatment must be carried out to remove the impurities in fly ash before zeolite synthesis. At present, there are various methods for preparing zeolites by applying fly ash, including hydrothermal synthesis, microwave irradiation, ultrasonic-assisted aging, seed crystallization, and so on [7,8,9]. Among them, the hydrothermal method is the most mature and commonly used method, while other methods are still in the laboratory research stage and have not yet met the requirements of industrial production. The commonly used pre-treatment to remove magnetic iron oxide from fly ash is magnetic separation, while for the removal of iron oxide and calcium oxide it is acid treatment, and for the effective removal of residual carbon from fly ash it is burning. These traditional impurity removal processes are relatively cumbersome and energy consuming, which causes huge costs in the pre-treatment process [10,11]. In addition, some other components will inevitably be brought out during the magnetic separation of iron oxide. Alkali-assisted hydrothermal treatment could improve the utilization of fly ash, but this process is time consuming. In order to improve the utilization efficiency of silicon and aluminum sources in fly ash and the purity of zeolite, zeolite was prepared through the molten alkali-assisted hydrothermal method in this paper. Due to the strong corrosive effect of alkalis, silicon oxide and aluminum oxide in fly ash can be efficiently and quickly extracted, while other impurities such as iron oxide and calcium oxide can be retained in the form of residues. Finally, the washed and dried residues can have some other applications such as secondary combustion and raw materials for other functional materials, once again proving the feasibility of alkali melting to extract silicon and aluminum from fly ash. In other words, the silicon and aluminum sources used for preparing zeolites from fly ash can be obtained through strong alkali melting combined with simple filtration [12,13].

The unique crystal structure of zeolite has shown excellent performance in the field of catalysis. In addition to typical industrial applications such as catalytic cracking [14,15], they have also played an important role in other fields, such as environmental treatment as adsorbents for treating atmospheric and water pollution [16,17]. With the advantages of large adsorption capacity, simple operation, and high processing efficiency, zeolite has been widely used for the removal of heavy metal from wastewater [18,19]. The removal of heavy metals in water by zeolite is achieved through a combination of ion exchange and adsorption, and is related to its own properties, influenced by multiple factors such as silicon–aluminum ratio, pore size, and its surface nature [20]. In general, adsorption, ion exchange and catalytic performace is very common to see in zeolites. In real life, zeolites have been applied in dehydration drying; for ethanol dehydration with low water content, zeolite adsorption dehydration is the optimal choice. Furthermore, the adsorption of H_2_S, SO_2_, NO_X_, and formaldehyde by zeolite can improve the air environment. In this report, the synthesized zeolites could also be used for ammonium adsorption. The ammonium-adsorbed zeolites can be used as fertilizers with slow-release ammonium for plant growth [21,22].

## 2. Results and Discussion

**Figure 1:**
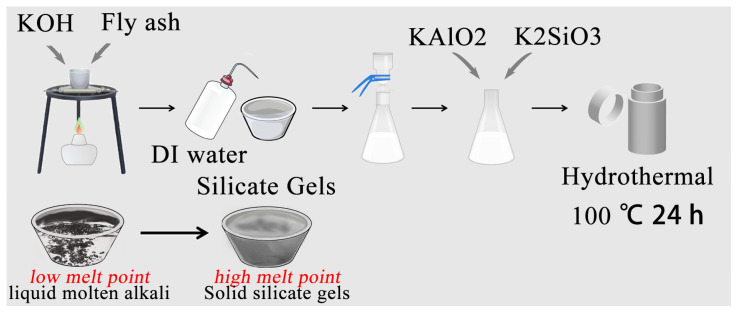
Schematic diagram of molten alkali-assisted preparation process.

**Figure 2:**
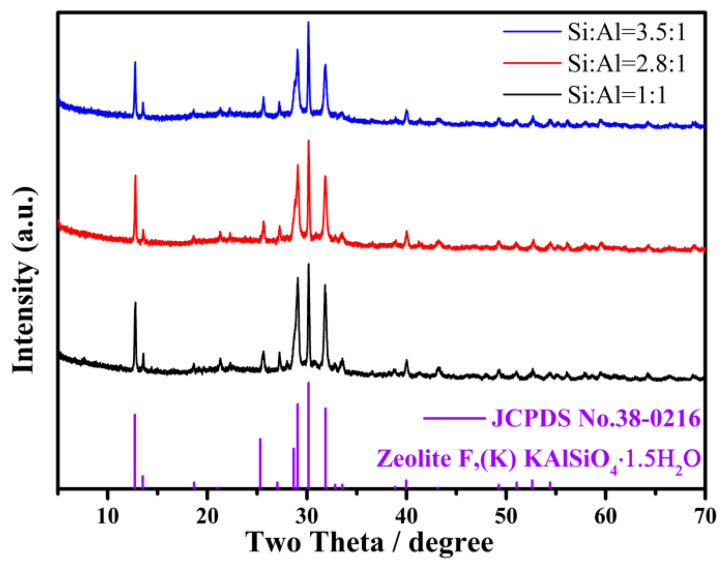
XRD patterns of obtained products from various ratios of Si to Al.

Strong alkalis, NaOH and KOH, were applied to extract silicon and aluminum from fly ash. Additional potassium silicate (sodium) or potassium aluminate (sodium) was added to adjust the target silicon–aluminum ratio (1:1, 2.8:1, and 3.5:1, respectively). The whole preparation process is depicted in Figure 1. The products obtained from crystallization at 100 °C for 24 h were analyzed by XRD, and the results are shown in Figure 2. The XRD diffraction spectrum of the obtained products matches well with the standard card JCPDS No.38-0216 and the crystal structure shows a tetragonal crystal structure. Similarly, it can be seen that there is no significant change in the diffraction spectrum (i.e., crystal configuration) of the product with an increasing silicon–aluminum ratio (from 1:1 to 2.8:1 and then to 3.5:1) [23]. It can be seen that in this reaction system, the crystal configuration of the product cannot be affected by simply changing the silicon–aluminum ratio, which may be due to the large amounts of base in the reaction system. It can be concluded that the strong base condition contributes to the fomation of a tetragonal crystal structure for zeolites.

It can be seen that the obtained zeolites prepared under any ratio of silicon to aluminum conditions have a uniform morphology. This also indicates that the zeolites can complete a good crystallization process in this reaction system, which is consistent with the experimental results of the XRD. In addition, as the silicon–aluminum ratio increases, the size of the product tends to decrease (the silicon–aluminum ratio of a, c, and e increases in sequence). This experimental result can be explained by the crystal growth theory. As the silicon–aluminum ratio increases, more [SiO_4_]^4−^ tetrahedrons can be formed in the reaction system to explode more crystal nuclei. In other words, it can provide more sites for crystal growth, and the size of the product will inevitably decrease without changing the concentration of other materials.

**Figure 3:**
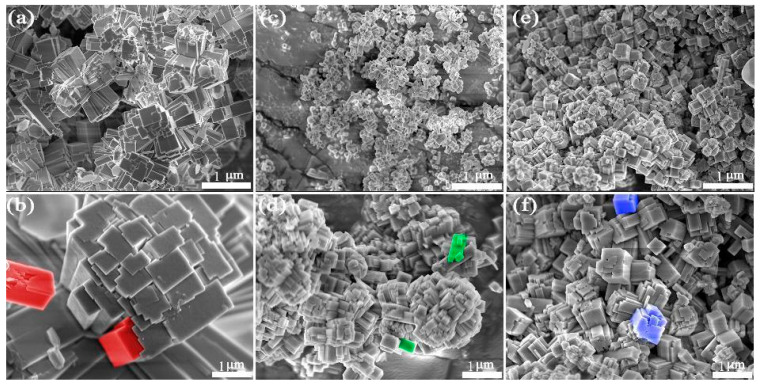
SEM images of zeolite-F from various ratios of Si to Al. (**a**,**b**) 1:1, (**c**,**d**) 2.8:1, and (**e**,**f**) 3.5:1.

Pseudo color processing (Figure 3 shows) on the high magnification SEM images (coloring the repeating units with bright colors) was performed to create a more intuitive display, and it can be clearly seen that the size of the zeolite crystal gradually decreases with the increasing ratio of silicon to aluminum. The crystal growth theory can provide a reasonable explanation for the experimental results. Due to increasing the silicon–aluminum ratio, the size of the product can become smaller, exposing more adsorption activity. Subsequent characterization is mainly based on the sample with the maximum silicon–aluminum ratio.

**Figure 4:**
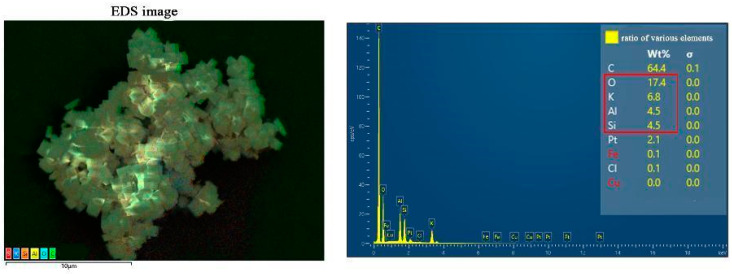
EDS results of zeolite-F obtained at the maximum silicon–aluminum ration.

As shown in Figure 4, the energy dispersion spectrum of the zeolite prepared under high silicon–aluminum ratio conditions indicates that the product contains main elements such as K, Si, Al, and O, etc. Among them, the highest content of C is mainly due to the background conductive adhesive used for testing, while Pt is the precious metal sprayed on the surface of the sample before testing to increase the conductivity of the sample. Other contents are as low as to be negligible. The experimental results demonstrate that the atomic ratio of K, Si, Al, and O in the molecular sieve we prepared is approximately 1:1:1:6. This result reveals that the obtained zeolite should belong to the type with a low ratio of silicon to aluminum [24,25].

**Figure 5:**
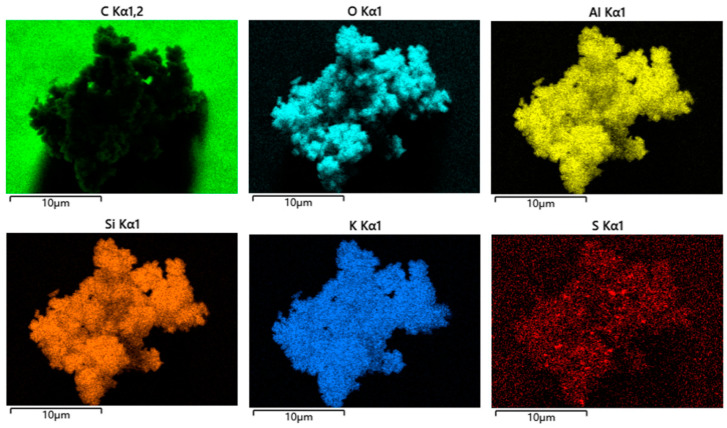
Mapping results of zeolite-F obtained at the maximum silicon–aluminum ration, every color represents one element ref to Figure 4.

In addition, we also conducted element distribution scanning (mapping test) on the sample, and the results are shown in Figure 5. The green background in the upper left corner is carbon, which is the background used during the test as a conductive adhesive; the red color in the lower right corner is S, indicating that the prepared zeolite contains a small amount of sulfur element, which is due to the sulfur content in the raw material fly ash. It also proves that the obtained product was prepared from fly ash as the raw material.

**Figure 6:**
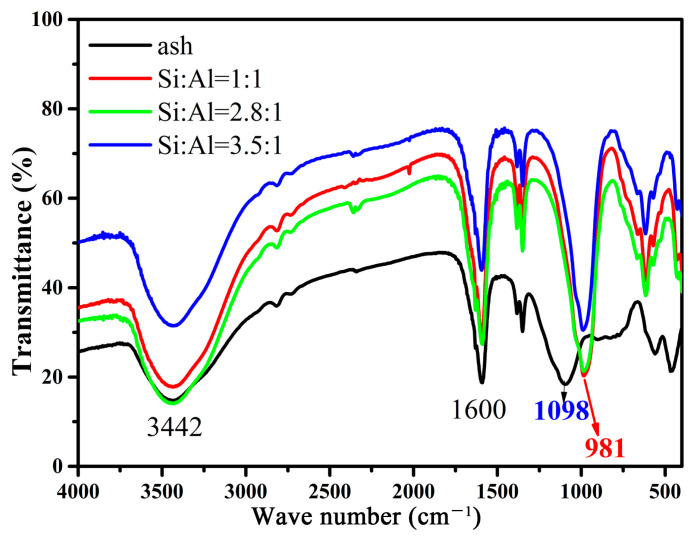
FTIR spectra of obtained products at various ratio of Si to Al and fly ash.

Figure 6 shows the FTIR spectra of fly ash and the synthetic zeolites. It can be seen that fly ash exhibits a strong -OH stretching vibration and -OH bending vibration at 3442 cm^−1^ and 1600 cm^−1^; the characteristic peak appearing at 1098 cm^−1^ is a Si-O-Si and Al-O-Si asymmetric stretching vibration. After the conversion of fly ash into zeolite-F, the infrared absorption peak undergoes a significant change, with the characteristic absorption peak at 1098 cm^−1^ shifting to 981 cm^−1^ [20,26,27]. This is because there is a large number of potassium ions in the structure of the zeolites, which inevitably leads to the existence of a Si-O-K skeleton. Compared to the Si-O-Si or Si-O-Al structures in fly ash, due to the larger atomic radius of K, the bond length of the Si-O-K structure in the moleculr is longer, resulting in a decrease in the wavenumber of the absorption peaks.

**Figure 7:**
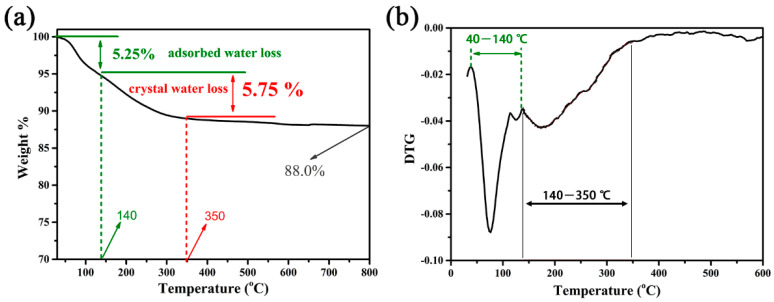
TG (**a**) and DTG (**b**) results of the obtained zeolite-F.

Gravimetric analysis of the synthesized zeolite was conducted to determine the content of crystalline water in the crystal structure of synthetic zeolite. The results are shown in Figure 7; there were two gradients of weight loss, which can be more clearly observed in the experimental results of first-order derivative DTG. Before 140 °C, the synthesized molecular sieve experienced a weight loss of 5.25%. The weight loss here is due to the presence of adsorbed water, including surface- and pore-adsorbed water. Continuing to increase the temperature, the synthesized molecular sieve continued to lose weight due to the detachment of crystalline water within the crystal structure, resulting in a weight loss of 5.75%. From this, it can be seen that the mass ratio of crystalline water in the synthetic zeolite reaches 6.1%. Based on the EDS spectrum data, the atomic ratio of K, Si, and Al obtained is 1:1:1, indicating that the molecular formula of the prepared molecular sieve is KSiAlO_4_·0.5H_2_O.

**Figure 8:**
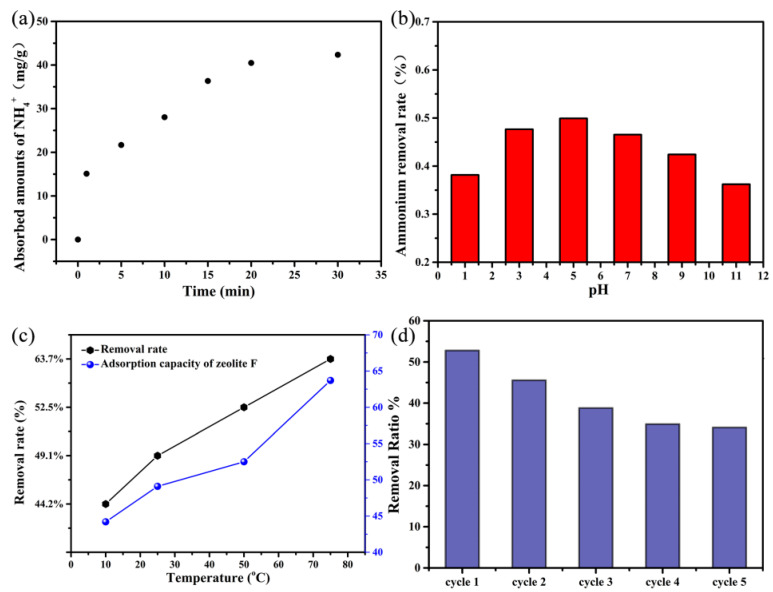
Influence factors time (**a**), pH (**b**), and T (**c**) on adsorption and (**d**) recylability.

The effect of adsorption time on the adsorption performance of ammonium ions was investigated. From Figure 8a, it can be seen that adsorption mainly occurs in the first 20 min. At the beginning of adsorption, the adsorption amount of ammonium increases rapidly. Afterwards, the adsorption rate of ammonium by zeolite significantly weakens, and the adsorption curve changes to be flattened. It can also be seen that the adsorption of the ammonia–nitrogen solution by the synthesized zeolite-F can basically reach adsorption equilibrium at 30 min. pH value is an important indicator in aqueous solutions, which not only affects the state of ammonium ions in water, but also affects the surface activity and electrical properties of adsorbents. This article investigates the effect of a solution pH range between 1.0 and 12.0 on the adsorption of ammonium ions on the obtained zeolite. As shown in Figure 8b, it can be seen that the adsorption capacity of synthetic zeolite for ammonia in water first increases and then decreases with pH from 1 to 11, showing good adsorption performance at pH = 5–6. This is because under strong acidic conditions, when the pH is below the isoelectric point of the [SiO_4_]^4−^ surface, the surface’s negative charge is weak, which affects the electrostatic adsorption of ammonium ions. When the pH value is too high, it can also affect the adsorption of ammonium ions. We believe that this may be due to the change in the form of ammonium ions under strong alkaline conditions, and the decrease in the concentration of free ammonium ions, which leads to a decrease in the removal rate of ammonium. Temperature is a crucial parameter for chemical reactions, as it not only affects the reaction rate but also the progress of the reaction. Therefore, the adsorption performance of synthetic zeolite for ammonium in water under conditions of 10, 25, 50, and 75 °C was investigated. As shown in Figure 8c, with the increase in temperature under 80 °C, the adsorption performance of the synthesized zeolite for ammonium is improved. As is well known, most adsorption reactions are exothermic, and experimental results show that heating is beneficial for adsorption. Therefore, we believe that the possible reason is that the interaction between ammonium ions and synthetic zeolite is mainly ion exchange, followed by electrostatic adsorption. Among them, the ion exchange between K^+^ and NH_4_^+^ in synthetic zeolite is an endothermic reaction. Furthermore, the recyclability of adsorption performance was also investigated. As shown in Figure 8d, after 5 cycles of reuse (recovery at 1 M KCl solution) [28,29,30], the ammonium adsorption performance of the obtained zeolite reaches a stable state and the ammonium removal rate reaches 34%. Compared with the first ammonium removal rate of 52%, it can be concluded that the ammonium exchange might occur just at the surface of the zeolite.

**Figure 9:**
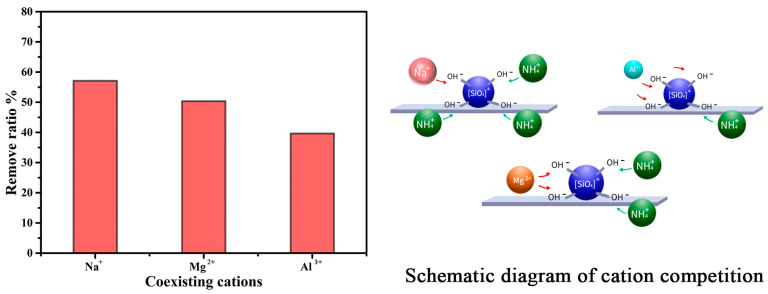
Influence of coexisting cations on the ammonium removal ratio.

As the ammonium removal is mainly caused by the ion exchange process of the obtained zeolite, the effect of the coexisting ions on the ammonium adsorption performance should be investigated. The cations with various positive charges such as Na^+^, Mg^2+^, and Al^3+^ were applied to illustrate the influencing mechanism. The result is shown in Figure 9; the ammonium removal performance reveals a decreasing trend when the number of positive charges increases. This result is consistent with the ion exchanging mechanism. Just as the schematic diagram of cations competition shows in Figure 9, the amount of surrounding ammonium decreases as the number of positive charges increases because the ion exchanging process obeys the law of charge equivalence.

## 3. Conclusions

During the pretreatment of fly ash, molten alkali was demonstrated to effectively extract Si and Al sources in fly ash. Without any adjusting of the pH of the reaction system, zeolite-F was successfully synthesized by the followed hydrothermal method, which reveals its super-convenient synthesis process. The whole process of fly ash treatment and zeolite synthesis was both energy- and time-saving, realizing the high-value usage of fly ash at the same time. The ammonium-adsorbed zeolites could also be recovered by a simple ion exchanging process. Furthermore, the ammonium-adsorbed zeolites might be used as an environmentally friendly ammonium fertilizer for agricultural plant growth.

## 4. Materials and Methods

### 4.1. Materials

The materials described in this report were all purchased from Aladdin (Shanghai, China). There was no need to purify before use. All the materials were Analytical Reagents. The purity of NaOH and KOH was larger than 95%. The fly ash was provided by Zhejiang ZhenengZhenhai Power Generation Co., Ltd (Ningbo, China). The detailed physical and chemical properties of the materials can be seen in Table 1.

### 4.2. Procedures of Zeolite Synthesis

#### 4.2.1. The Formation of Silicate Gels

Calculated amounts of NaOH and KOH were added into fly ash (mass ratio was 10:1; 10 g alkali and 1 g fly ash) and the mixed powders were placed into a nickel crucible. An alcohol lamp was applied to heat the mixed powder. The NaOH and KOH soon became molten, which is beneficial to extract SiO_2_ and Al_2_O_3_ because of its strong corrosiveness. The molten alkali reacted with SiO_2_ and Al_2_O_3_ quickly and resulted in the production of K_2_SiO_3_ and KAlO_2_, which could not be dissolved in molten alkali because of their higher molten points. Thus, some white precipitate was separated out from the transparent molten alkali system. The alkali silicate gels were formed with the extraction process and this extraction process was maintained for around another 30 min so as to fully extract the SiO_2_ and Al_2_O_3_ from the fly ash. The rapid formation of alkali silicate gels further reveals that the extraction of SiO_2_ and Al_2_O_3_ from fly ash by molten alkali treatment was time- and energy-saving.

#### 4.2.2. Hydrothermal Synthesis of Zeolite-F

When the silicate gels mentioned above cooled down to room temperature, deionized water (DI water) was added to dissolve the soluble composites such as K_2_SiO_3_ and KAlO_2_. A filtration process was conducted to separate the residual solid product (Fe_2_O_3_, CaO, and C, etc.) and the solution containing K_2_SiO_3_ and KAlO_2_. Finally, a calculated Si and Al source was added to the solution to regulate the appropriate ratio of Si to Al (the ratio of Si to Al ranged from 1:1 to 3.5:1). The followed hydrothermal process was performed at 100 °C for 24 h. When cooled down to room temperature, white precipitate was formed at the bottom of teflon lining. DI water and ethanol was used to wash the surface residue chemicals by centrifugation several times. The product was finally dried at 60 °C in the vacuum oven.

#### 4.2.3. Experiment of Ammonium Adsorption

The volume of ammonium in this experiment was 50 mL (concentration was set as 100 mg/L), which was placed in a conical flask with a capacity of 250 mL. Then, 50 mg of synthetic zeolite was added and shaken in a shaker. The temperature of the shaker was set at 25 °C and the speed was 200 rpm. An amount of 50 mg of zeolite was added to the solution, and after adsorption saturation, the supernatant was quickly centrifuged. Then, a glass fiber filter membrane with a pore size of 0.22 μm was used to filter the supernatant, and Nessler’s reagent was used to color the filtration. Lasty, a spectrophotometer was used to measure the absorbance after the coloring procedure. The absorbance of the filtration was determined by the concentration of ammonium. The cyclic adsorption performance test of zeolite was conducted the same way as mentioned above.

### 4.3. Characterization

The XRD patterns were recorded by using PANalytical B.V. Empyrean X-ray powder diffraction (Malvern Panalytical, Enigma Business Park, Malvern, UK) with Cu Kα radiation over a range of 10–70° (2θ) with 0.02° per step. SEM images were obtained with a JSM-6700F electron microscope (JEOL, 1-2 Musashino 3-chome, Showa City, Tokyo, Japan). The thermogravimetric analysis (TGA) was performed by using a Netzch Sta 449c (Netzch Company, Selb, Germany) thermal analyzer system at a heating rate of 10 °C/min in air. The FTIR spectrum was recorded by Fourier Transform Infrared Spectrometry FTIR-650.

## Figures and Tables

**Table 1:** Physical and chemical properties of materials.

Materials	Melt Point/°C	Solubility/	Source
NaOH	318 (low)	strong corrosive	Aladdin
KOH	361 (low)	strong corrosive	Aladdin
Na_2_SiO_3_	1089 (high)	water soluble	Aladdin
NaAlO_2_	1650 (high)	water soluble	Aladdin
K_2_SiO_3_	976 (high)	water soluble	Aladdin
Fly ash	/	water insoluble	SiO_2_ 48%, Al_2_O_3_ 33%

## Data Availability

The data presented in this study are openly available in article.
